# Computer-aided designing of a novel multi‑epitope DNA vaccine against severe fever with thrombocytopenia syndrome virus

**DOI:** 10.1186/s12879-024-09361-6

**Published:** 2024-05-07

**Authors:** Yiran Tao, Yu Zhang, Yumeng Li, Qiao Liu, Jin Zhu, Minjun Ji, Gaoqian Feng, Zhipeng Xu

**Affiliations:** 1 Department of Pathogen Biology, National Vaccine Innovation Platform, School of Basic Medical Sciences, Nanjing Medical University, Nanjing, People’s Republic of China; 2 NHC Key Laboratory of Antibody Technique, Nanjing Medical University, Nanjing, People’s Republic of China; 3 Department of Chronic Communicable Disease, Center for Disease Control and Prevention of Jiangsu Province, Nanjing, People’s Republic of China; 4 Huadong Medical Institute of Biotechniques, Nanjing, People’s Republic of China; 5 The First Clinical Medical College of Nanjing Medical University, Nanjing, People’s Republic of China

**Keywords:** Severe fever with thrombocytopenia syndrome, DNA vaccine, Bioinformatics, Cellular immune responses, pVAX1

## Abstract

Severe fever with thrombocytopenia syndrome (SFTS) is an emerging tick-borne viral disease caused by the SFTS virus (*Dabie bandavirus*), which has become a substantial risk to public health. No specific treatment is available now, that calls for an effective vaccine. Given this, we aimed to develop a multi-epitope DNA vaccine through the help of bioinformatics. The final DNA vaccine was inserted into a special plasmid vector pVAX1, consisting of CD8^+^ T cell epitopes, CD4^+^ T cell epitopes and B cell epitopes (six epitopes each) screened from four genome-encoded proteins——nuclear protein (NP), glycoprotein (GP), RNA-dependent RNA polymerase (RdRp), as well as nonstructural protein (NSs). To ascertain if the predicted structure would be stable and successful in preventing infection, an immunological simulation was run on it. In conclusion, we designed a multi-epitope DNA vaccine that is expected to be effective against *Dabie bandavirus*, but in vivo trials are needed to verify this claim.

## Introduction

Emerging zoonoses are major and global challenges for public health [1]. Severe fever with thrombocytopenia syndrome (SFTS) is an emerging zoonosis caused by tick-borne SFTS virus (*Dabie bandavirus*), which was initially reported in China [2] and is prevalent at the moment in East Asia and has a high mortality rate of 12–50% [3, 4]. High fever and thrombocytopenia are the typical clinical signs of *Dabie bandavirus* infection. Additional symptoms include gastrointestinal problems, leukopenia, hemorrhagic tendencies, and others [5, 6]. The three stages of the SFTS clinical course include the high fever stage, multiple organ dysfunction (MOD) stage, and recovery stage [7]. Within five days of the disease’s onset, it will have advanced to the most serious MOD stage. Clinical signs of this stage include multi-organ failure, disseminated intravascular coagulation (DIC), persistently low platelet counts, neurological complaints, and bleeding manifestations [5]. Ribavirin with broad-spectrum antiviral activity, favipiravir targeting RNA-dependent RNA polymerase, and calcium channel inhibitors have been selected as treatments for SFTS in the expert consensus released in 2022 in China [8]. Among the three drugs, calcium channel inhibitors have the most potential for clinical application, since a Chinese clinical survey showed that nifedipine can reduce the mortality rate of *Dabie bandavirus* -infected patients by more than five times [9]. Still, there is no effective preventive method against this disease, this calls for a preventive vaccine that is reliable, long-lasting, low-cost, and secure, which is a permanent need in the battle against conventional, emerging and re-emerging infectious diseases [10]. Yu, *et al*. [11] reported and assessed two live attenuated viruses as vaccine candidates in ferret model. Dong, *et al*. [12] developed a live attenuated recombinant vesicular stomatitis virus-based vaccine candidate expressing the SFTSV Gn/Gc glycoproteins (rVSV-SFTSV/AH12-GP). Moreover, Kim, *et al*. [13] developed an mRNA-Gn vaccine administrated in lipid nanoparticle encapsule which successfully induced neutralizing antibodies and T-cell responses in mice. Notably, Kwak, *et al*. [14] have developed several DNA vaccines based on the genome of the virus.

Compared with traditional vaccines such as live attenuated vaccines, DNA vaccines have numerous potential advantages including inherent safety and a more rapid production time [15]. Plasmids with the desired antigen’s encoding genes are used to create DNA vaccines, which would be expressed in the host and induce the immune response [16, 17]. In the field of creating DNA vaccines, advances in immunoinformatics, bioinformatics and reverse vaccinology pipelines are increasingly widely applied [18]. These technologies were used to screen potential epitopes which include the conformational and linear epitopes that could be recognized by the immune system [19]. The genome of the *Dabie bandavirus* encodes four proteins, nuclear protein (NP), glycoprotein (GP), RNA-dependent RNA polymerase (RdRp), and a non-structural protein (NSs). GP is cleaved during synthesis into Gn and Gc [20]. Based on Gn/Gc, Kwak JE, *et al*. have developed a DNA vaccine [14]. The fact that numerous human monoclonal antibodies generated by SFTS patients recognized NP further indicated that the NP was important for humoral responses to *Dabie bandavirus* infection [21]. For NSs, it has been found that it could induce pro-viral autophagy by interacting with mTOR, where autophagy enhances *Dabie bandavirus* infection and propagation [22].

Based on the four proteins listed above, the goal of this study was to create a multi-epitope DNA vaccine against *Dabie bandavirus*. We assembled a vaccine containing high-potential CTL, HTL and B cells epitopes, and assessed the vaccine’s stability and effectiveness, providing a possible way to prevent *Dabie bandavirus* infection.

## Methods

### Sequence and structure retrieval

**Fig. 1:**
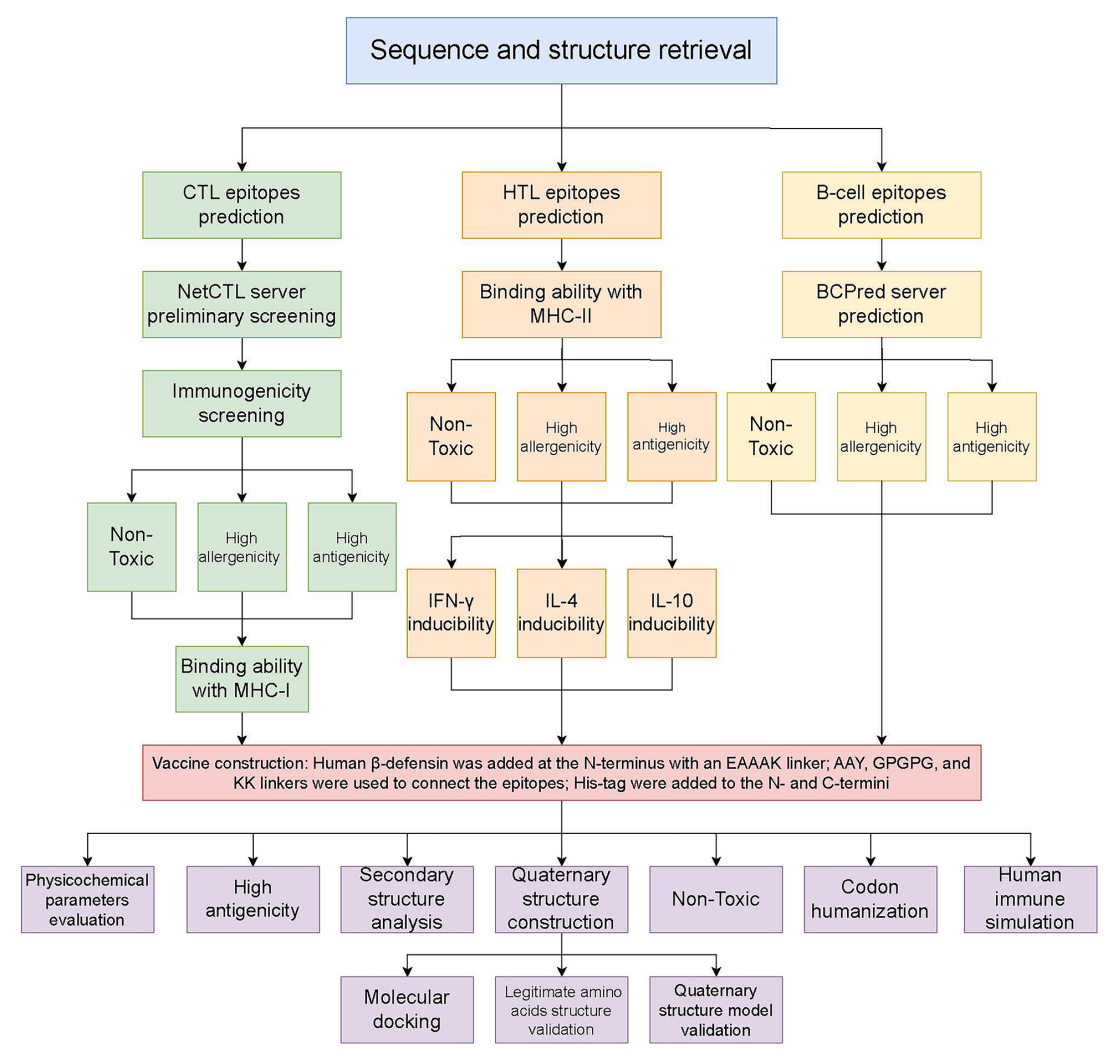
The general workflow of the study

Details of the workflow are given in Fig. 1. We selected all four proteins encoded by the SFTS genome and the sequences were retrieved from GenBank (https://www.ncbi.nlm.nih.gov/genbank/), including glycoprotein (ID: AWW14922.1), RNA-dependent RNA polymerase (RdRp, ID: AWW14921.1), nucleoprotein (NP, ID: AVM39051.1) and nonstructural protein (NSs, ID: AVM39050.1).

### CTL epitopes prediction

NetCTL 1.2 (https://services.healthtech.dtu.dk/services/NetCTL-1.2/) was used to predict the CTL epitopes, using all the server-provided HLA supertypes, and the threshold of prediction score was 1.0. Then the immunogenicity of the epitopes was confirmed by the MHC-I Immunogenicity IEDB tool (http://tools.iedb.org/immunogenicity/). The MHC-I binding ability prediction was conducted by the IEDB MHC-I binding prediction tool (http://tools.iedb.org/mhci/), ensuring that the percentile rank < 2. Toxicity and allergenicity of the epitopes were checked by ToxinPred (https://webs.iiitd.edu.in/raghava/toxinpred/multi_submit.php) and AllerTOP 2.0 (https://www.ddg-pharmfac.net/AllerTOP/) respectively. The antigenicity of the epitopes was calculated using the VaxiJen server (http://www.ddg-pharmfac.net/vaxijen/VaxiJen/VaxiJen.html).

### HTL epitopes prediction

The IEDB MHC-II binding predictions tool (http://tools.iedb.org/mhcii/) was used to design HTL epitopes. A percentile rank < 0.25 was set as the threshold. The inducibility of interferon-γ (IFN-γ), interleukin-4 (IL-4), and interleukin-10 (IL-10) were conducted by IFNepitope (https://webs.iiitd.edu.in/raghava/ifnepitope/), IL10Pred (https://webs.iiitd.edu.in/raghava/il10pred/), and IL4Pred (https://webs.iiitd.edu.in/raghava/il4pred/index.php). We comprehensively considered the cytokine-inducing results in the final selection of epitopes. The toxicity, allergenicity and antigenicity were checked then.

### B-cell epitopes prediction and selection

B-cell epitopes play an important role in B cell-mediated humoral immune response. Thus, B-cell epitopes up to standard should be selected as part of the multi-epitopes’ vaccine. Firstly, BCpred server was employed to predict the B-cell epitopes of the selected protein. Secondly, Vaxijen was used to perform antigenicity analysis on the B-cell epitopes predicted by BCpred server. As potential B-cell epitope candidates, six of the most antigenic epitopes were chosen. The toxicity and allergenicity of six candidate epitopes were further tested by ToxinPred and AllerCatPro 2.0.

### Construction of the multi-epitope vaccine

**Fig. 2:**
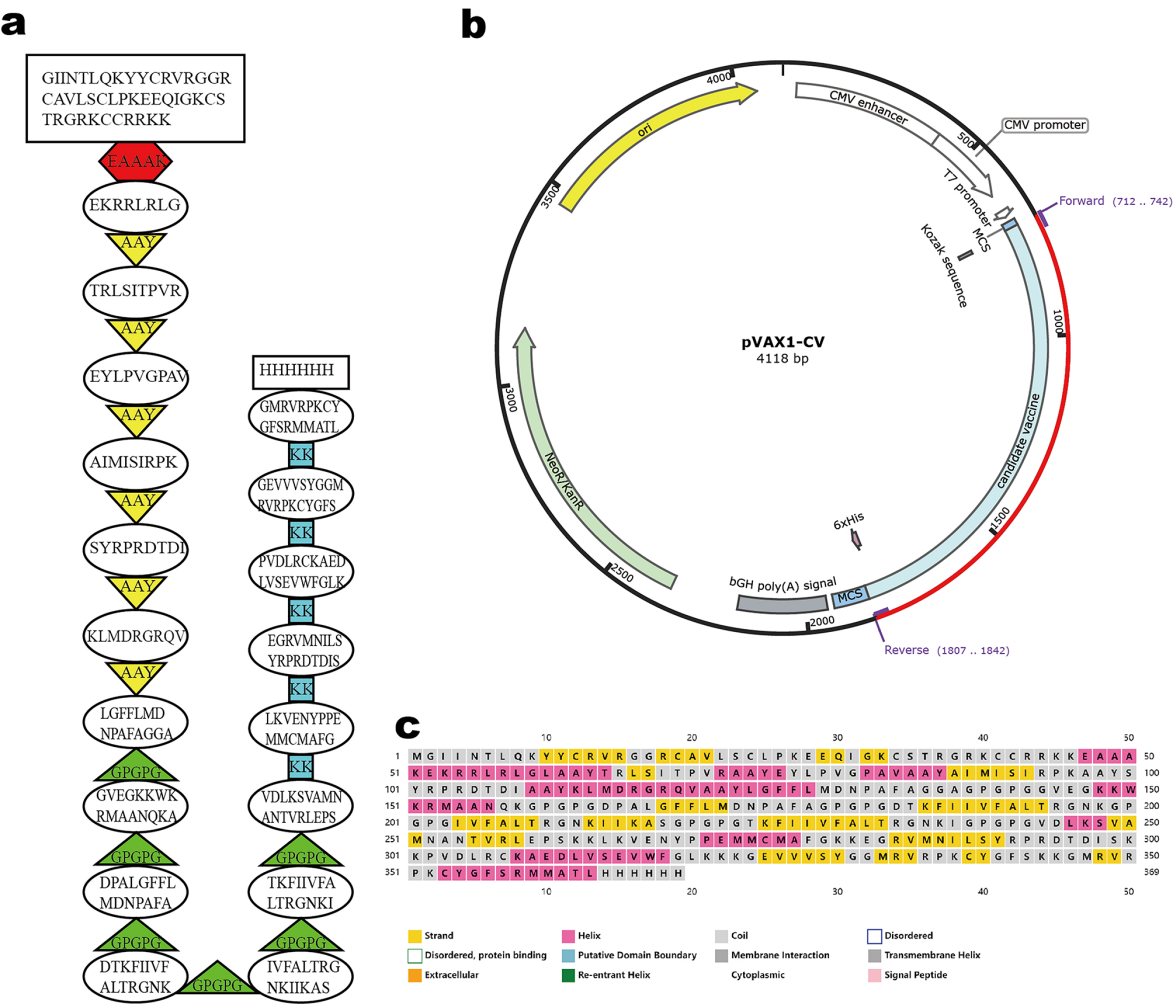
**(a)** The final epitopes for DNA vaccine and the linkers. **(b)** The DNA sequence in pVAX1 vector. **(c)** The predicted secondary structure

Different linkers, including AAY, GPGPG, and KK, were used to connect the epitopes. Human β-defensin-3 was added at the N-terminus with an EAAAK linker in order to enhance the immune response. At last, a methionine and a His-tag were added to the N- and C-termini, respectively (Fig. 2a). The cDNA of the vaccine was generated using the Java Codon Adaptation Tool (http://www.jcat.de/), then the Kozak sequence was added for initiation of replication. The cDNA was inserted between HindIII and EcoRI restriction sites of the pVAX1 vector, which was specially designed for DNA vaccine development (Fig. 2b).

### Homology modeling and validation

We used I-TASSER server (https://zhanggroup.org/I-TASSER/) to predict the 3D structure of the vaccine. The obtained homology models were then refined by the GalaxyRefine tools (https://galaxy.seoklab.org/cgi-bin/submit.cgi?type=REFINE). The refined structures were analyzed by the SAVES 6 server (https://saves.mbi.ucla.edu/) and ProSA web (https://prosa.services.came.sbg.ac.at/prosa.php) and the structure with the best results synthetically was further analyzed.

### Physiochemical properties and immune properties prediction

A flexible and powerful server, ProtParam server could help us predict the Physiochemical properties of the vaccine more easily. In addition, to confirm that the constructed vaccine can indeed produce immunological effects, we analyzed the antigenicity of the vaccine by Vaxijen and AntigenPro, and predicted the allergenicity of the vaccine by AllerTOP2.0 network tool. Finally, the designed vaccine was compared to human proteins using NCBI’s BLASTp tool to ensure that the immune system could be properly activated.

### Molecular interaction analysis of the vaccine with TLR-3

HDOCK server (http://hdock.phys.hust.edu.cn/) carried out the molecular docking experiments of the vaccine with Toll-like receptor 3 (TLR-3). The PyMol was used to generate the final images. In-depth knowledge regarding the relationships between vaccine-receptor binding was obtained using the PDBsum server. In order to gain a better understanding of the vaccine-TLR3 complex’s dynamic stability, the final stage required putting it to molecular modeling. For the complex, we ran 10ns of simulation using the AMBER20 tool. We applied the MM/GBSA method to evaluate the binding free energies inside the complex. This platform predicted and analyzed the protein-protein complex using MM/GBSA and computational docking.

### Human immune simulation

To mimic the immunological response following the administration of the vaccine, C-ImmSim sever (https://kraken.iac.rm.cnr.it/C-IMMSIM/) was utilized [23–25]. All of the simulation’s default settings were applied.

## Results

### CTL epitopes prediction

CTL epitopes were screened by using the NetCTL 1.2 server. These epitopes were shortlisted based on the MHC-I binding affinity, C-terminal cleavage affinity and TAP transport efficiency. Epitopes were further analyzed, including their immunogenicity, toxicity, antigenicity, etc. Taking all the analysis results into consideration, 6 epitopes with the highest percentile ranks from the four proteins (GP, RdRp, NP, NSs) were finally selected (Table 1).

### HTL epitopes prediction

The IEDB MHC-II binding predictions tool’s percentile rank (< 0.25) and its capacity to induce cytokines, particularly IFN-γ, were used to identify the HTL epitopes. Six epitopes from RdRp and NP met the rules (Table 2). The antigenicity, toxicity and allergenicity were also noted in Table 2.

### Linear B-cell epitopes prediction and selection

High antigenicity is a crucial factor to consider when choosing B-cell epitopes to create an effective multi-epitope vaccine. Therefore, B-cell epitopes with 16,18,20-mer lengths, predicted by the BCPred server (Threshold > 0.8), were ordered from highest to lowest antigenicity. Six of the most antigenic epitopes were selected for the final vaccine. Additionally, these six B-cell epitopes were confirmed to be non-toxic and non-allergic using ToxinPred and AllerCatPro 2.0, respectively. The six B-cell epitopes chosen for further analysis was shown in Table 3.

**Table 1:** List of final CTL epitopes and detailed information

Protein	Peptide sequence	MHC binding affinity	C-terminal cleavage affinity	TAP transport efficiency	Prediction score	Immunogenicity	Toxicity	Allergenicity	Antigenicity score (> 0.5)	MHC-I binding prediction
NSs										
	EKRRLRLGL	0.3325	0.1229	0.858	1.1969	0.11724	None	None	1.5340	< 2
NP										
	TRLSITPVR	0.3769	0.7224	1.521	1.1863	0.01083	None	None	2.1836	< 2
	EYLPVGPAV	0.5025	0.8077	0.494	1.2159	0.07084	None	None	1.4436	< 2
RdRp										
	AIMISIRPK	0.528	0.1913	0.856	1.0653	0.0783	None	None	2.6252	< 2
	SYRPRDTDI	0.3845	0.9207	0.949	1.0043	0.12264	None	None	2.4142	< 2
GP										
	KLMDRGRQV	0.6154	0.7269	0.476	1.0502	0.02362	None	None	1.3673	< 2

**Table 2:** L﻿ist of final HTL epitopes and detailed information

Protein	Peptide sequence	Method		Percentile rank(<0.25)	Antigenicity score (> 0.5)	Toxicity	Allergenicity	IFN-γ	IL-4	IL-10
RdRp										
	LGFFLMDNPAFAGGA	NetMHCIIpan		0.11	0.5485	None	None	Positive	-	Inducer
	GVEGKKWKRMAANQK	Consensus (smm/nn/sturniolo)		0.21	0.6958	None	None	Positive	Inducer	Inducer
	DPALGFFLMDNPAFA	Consensus (comb.lib./smm/nn)		0.15	0.5923	None	None	Positive	Inducer	-
NP										
	DTKFIIVFALTRGNK	Consensus (smm/nn/sturniolo)		0.04	0.7901	None	None	Negative	Inducer	Inducer
	IVFALTRGNKIIKAS	Consensus (comb.lib./smm/nn)		0.01	0.715	None	None	Negative	Inducer	Inducer
	TKFIIVFALTRGNKI	Consensus (comb.lib./smm/nn)		0.01	0.5124	None	None	Negative	Inducer	Inducer

**Table 3:** List of final B-cell epitopes

Protein	Peptide sequence	Prediction Score (> 0.8)	Antigenicity score (> 0.5)
NSs			
	VDLKSVAMNANTVRLEPS	0.82	1.0716
NP			
	LKVENYPPEMMCMAFG	0.87	1.6071
RdRp			
	EGRVMNILSYRPRDTDIS	0.82	1.4985
	PVDLRCKAEDLVSEVWFGLK	0.81	1.4377
Gly			
	GEVVVSYGGMRVRPKCYGFS	0.87	1.8168
	GMRVRPKCYGFSRMMATL	0.83	1.7512

### Physicochemical parameters evaluation

The physiochemical properties of the final vaccine were computed using the ProtParam service, which is often regarded as the initial phase of built amino sequence assessment. The results showed that the designed vaccine’s molecular weight was 40434.68 g/mol with the theoretical Isoelectric point (pI) of 10.19. It has 25 negatively and 67 positively charged residues in the vaccine. This candidate vaccine’s Instability index was computed to be 24.91, indicating that the designed vaccine had good stability in different temperatures (instability index < 40). At the same time, the vaccine’s estimated half-life differed in mammalian reticulocytes (in vitro), yeast (in vivo) and *Escherichia coli* (in vivo), where it took 30 h, more than 20 h and more than 10 h to reach its certain concentration. In addition, the aliphatic index is a critical parameter to be evaluated because high aliphatic index is associated with thermal stability in usual. The final vaccine construct could be regarded as a thermostable protein for its aliphatic index 71.95. Last but not least, the grand average of hydropathicity (GRAVY) of this vaccine construct was − 0.314, classifying the protein as hydrophilic. The specific value was illustrated in Table 4.

**Table 4:** The physicochemical properties of the *Dabie bandavirus* candidate vaccine evaluated by ProtScale server

	Number of amino acids	Molecular weight	Theoretical pI	Instability index(< 40)	Aliphatic index	GRAVY
Recombinant Vaccine	369	40434.68	10.19	24.91	71.95	-0.314

**Table 5:** Binding free energy of the vaccine-TLR-3 complex

	VDW	ELE	GB	SA	TOTAL
Complex	-94.96	-2775.07	2828.91	-12.54	-53.66

### Antigenicity and allergenicity prediction

Vaccine antigenicity was estimated using the Vaxijen server to ensure it could stimulate protective humoral immune responses. The result indicated that the vaccine was a potential antigen with a high antigenicity score of 0.9018, setting 0.4 as the threshold. Besides, the allergenicity of the vaccine needs to be predicted to avoid unnecessary hypersensitivity. The AllerTOP v.2.0 server’s anticipated outcome showed that the candidate vaccination was classified as non-allergen (not shown in the figures).

### Homology modeling and validation

**Fig. 3:**
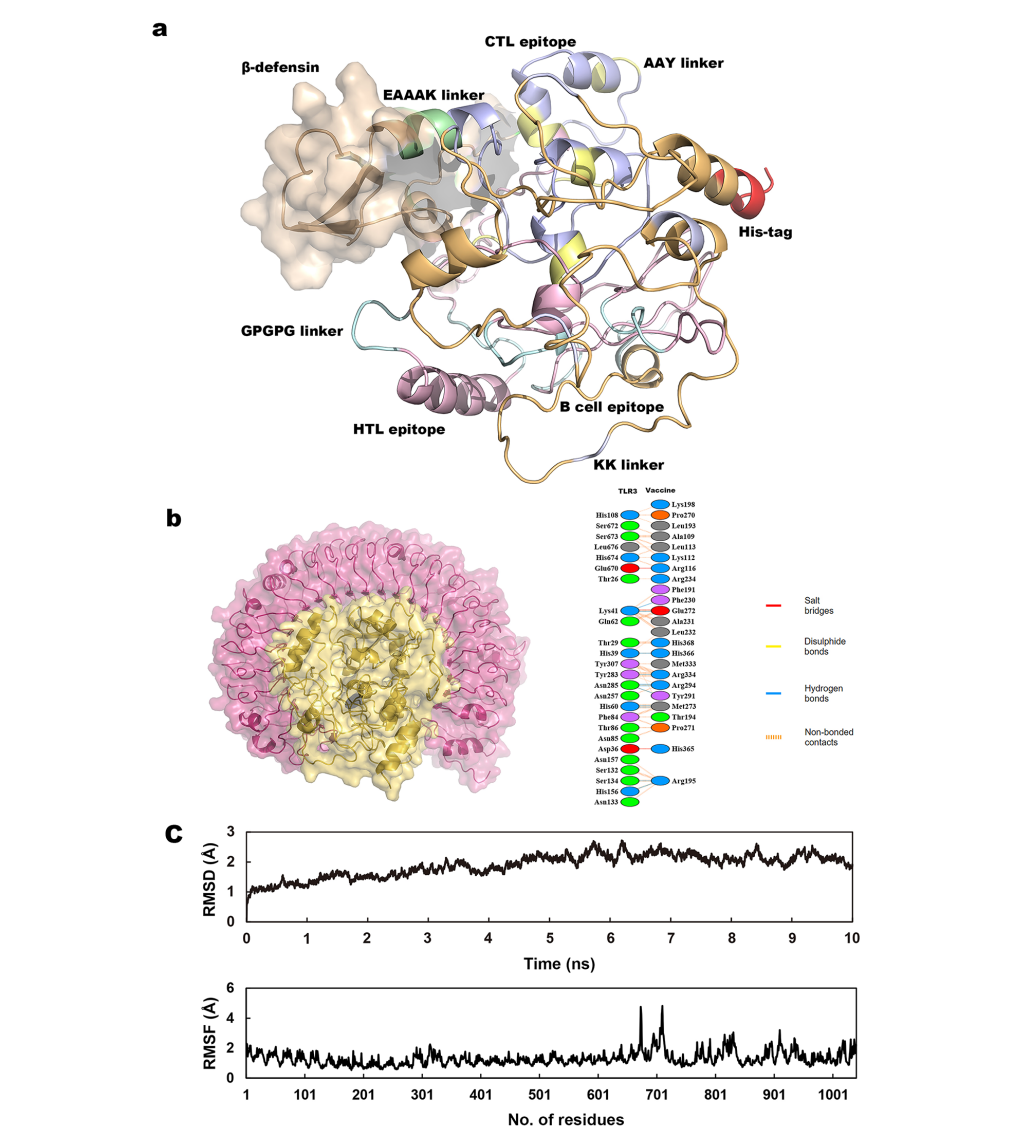
**(a)** The fined 3D structure of the final product. **(b)** The complex of the vaccine with TLR-3. **(c)** The molecular dynamics simulations of the vaccine with TLR-3

**Fig. 4:**
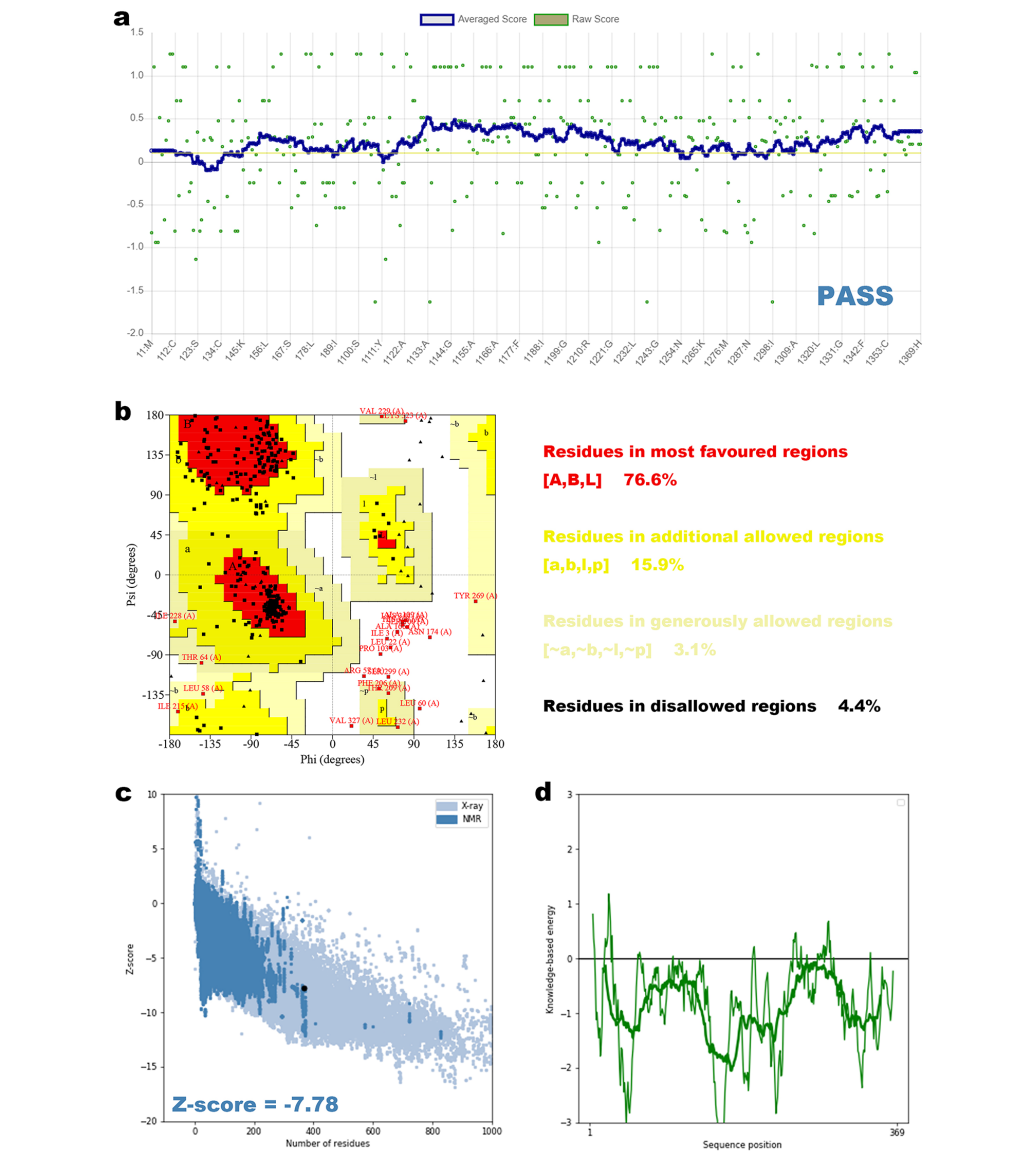
**(a)** Verify3D confirmed a valid structure of the final product. **(b)** Validation of the final structure by a Ramachandran plot. **(c and d)** ProSA-web Z-score plot and local model quality for the 3D structure

The I-TASSER server generated five models of the 3D structures, after further analyses, the refined model 3 (Fig. 3a) was selected for further study. The Ramachandran plot was performed by the SAVES 6 server (Fig. 4b), the result shows that 76.6% of the residues were in the most favored regions, 15.9% were in additional allowed regions, 3.1% were in generously allowed regions, with only 4.4% residues in disallowed regions. More than 80% of the amino acids scored higher than 0.2 in the 3D/1D profile utilizing, according to Verify3D, validating a legitimate structure (Fig. 4a). To identify any potential model flaws, ProSA-web additionally assessed the refined structure. The Z-score was − 7.78 (Fig. 4c and d).

### Molecular interaction analysis of the vaccine with TLR-3

HDOCK predicted 10 complexes for the complexes of the vaccine with TLR-3 and ranked them beyond the docking energy score (the lower the energy score, the higher the rank). We selected the structure with the highest rank and used Pymol to generate the 3D structures (Fig. 3b and c). The PDBsum server was used to further show the protein-protein interactions. The results revealed the hydrogen bonds and non-bond interactions between the vaccine and TLR-3. 11 H-bonds and 164 non-bond interactions were predicted in the complex with TLR-3 (Fig. 3b). Molecular simulation of the vaccines-TLR3 complexes demonstrated a stable dynamic behavior during the simulation (Fig. 3c), the RMSD of each complex stabilized at 1.9 Å following a uniform pattern then until 10ns with no significant deviation revealing that the vaccine binds stably to the TLR-3. On the other hand, the RMSF (1.3 Å on average) of the complex as shown in Fig. 3c is within acceptable range. The binding free energies of the vaccine with TLR-3 was shown in Table 5.

### Human immune simulation

**Fig. 5:**
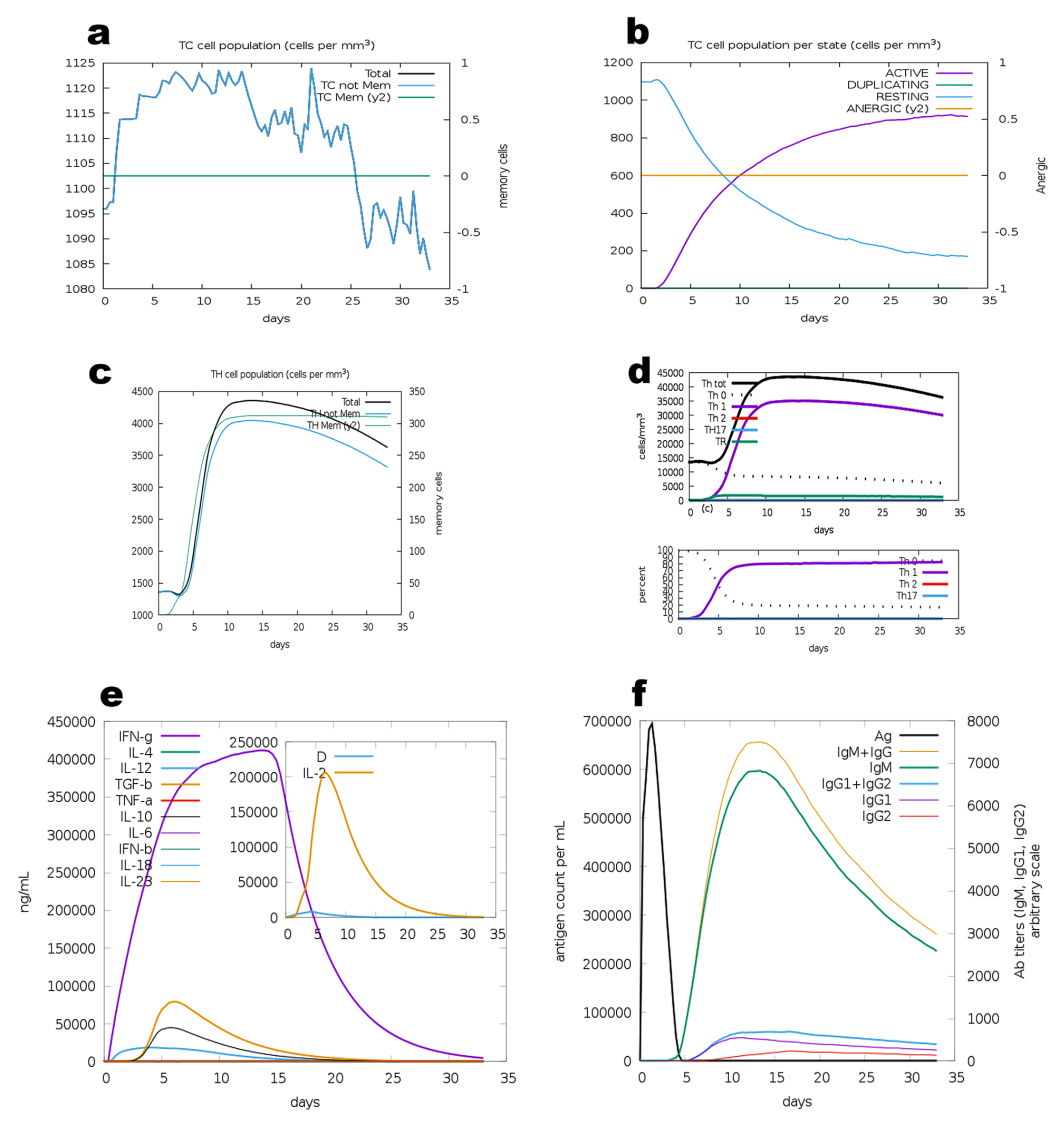
Immune simulation of the vaccine. **(a and b**) The change in total TC cell population and population per state. **(c and d)** The change in total TH cell population and population per state. **(e)** The changes in the secretion of cytokines. **(f)** The changes in the secretion of antibodies

The C-ImmSim server modeled the immunological response to the final vaccine design. A sharp increase in Tc (cytotoxic) cell population was observed after the injection (Fig. 5a), with the number of active Tc cells keeping rising (Fig. 5b). The Th (helper) cell was also responsive to the vaccine (Fig. 5c). In addition, Th1 cells were activated after the injection (Fig. 5d), under this, a significant increase in IFN-γ could be observed (Fig. 5e). A significant antibody response was also observed (Fig. 5f).

## Discussion

Since its initial identification in China in 2009, SFTS has become an emerging infectious disease and a substantial risk to public health, with a steadily expanding geographic dispersion, especially in China, Japan and Korea [2, 26, 27]. Up until December 2020, epidemiological data showed that there were 13,305 confirmed cases of SFTS in 24 Chinese provinces, and that the incidence was rising annually [28, 29]. In addition, the *Dabie bandavirus* is able to rapidly evolve and enhance genetic diversity, which has garnered significant attention already [30]. Worse still, apart from preventing tick bites, there is no specific treatment for SFTS available [31]. The creation of protective vaccinations is therefore becoming a major problem and preparedness strategies.

With the availability of large-scale sequencing data in well-organized databases and the advancement in sequencing techniques, reverse vaccinology places a strong emphasis on developing vaccines through a genome-based approach [32]. Bioinformatics procedures have played essential roles in designing more robust vaccines, which have saved a great deal of time and without the necessity to manipulate a pathogenic microorganism in vitro [33]. Furthermore, in comparison to mRNA vaccines and other conventional vaccine strategies, DNA vaccines may have excellent prophylactic potential since they can rapidly modify sequences to adapt to the emergence of new diseases [34]. Besides this, the strength of DNA vaccines such as the simplicity of preparation and storage are becoming more obvious and popular [35]. In the past few years, the multiple epitope-based vaccine design approach has become feasible and efficient, which can enhance the safety and effectiveness of vaccines [36, 37]. Therefore, the accurate epitope prediction for both T cells and B cells of a special genome-encoded protein plays a crucial role in the properties of multi-epitope DNA vaccines [38]. Up to now, the efficacy and safety of multi-epitope DNA vaccines have been verified through human clinical trials [39]. For instance, the first genetic vaccination to combat the COVID-19 pandemic was licensed [40].

In this study, we used bioinformatics methods to predict the CTL, HTL and B-cell epitopes of *Dabie bandavirus*’s genome-encode proteins and constructed a multi-epitope DNA vaccine via *in silico* genomic databases. Additionally, high antigenicity and immunogenicity of the vaccine have been confirmed using bioinformatics resources available online, including VaxiJen and IEDB. Instead of using the full-length proteins, we selected several high-potential epitopes from each protein to obtain a reduced size of the final products, which means the prevention of the possibility of insolubleness due to the large molecular weight [41]. The screening of dominant epitopes made the most use of the immunogenicity of the antigen and evaluated the toxicity and allergenicity, and both the CTL, HTL and B-cell epitopes were screened separately and combined in the multi-epitope construction to expand the width of immune response and ensure safety. For CTL and HTL epitopes, they all have a good binding ability with MHC-I/MHC-II, which is predicted by online services. Meanwhile, for HTL, the inducibility of IFN-γ [42], IL-4 [43] and IL-10 [44] was taken in consideration [45]. As a result, this multi-epitope DNA vaccine had a substantial advantage over currently employed vaccinations in that its capability to stimulate both humoral and cellular immune responses [46].

The four proteins (GP, NP, RdRp, NSs) encoded by the genome of the *Dabie bandavirus* play crucial roles in the infection and propagation process of the virus [47]. Therefore, in this study, candidate epitopes were screened from all four proteins, which was also strategically different from other vaccine designs such as the one Suleman M, *et al*. developed [48]. Suleman M, *et al*. selected dominant epitopes only from RdRp and GP beyond the antigenic score, while we not only focused on the antigenicity but also attempted to stimulate the immune system against *Dabie bandavirus* in the whole process of *Dabie bandavirus* infection. After the preliminary designing, we conducted a homology modeling and validated that the target post-transcriptional product was structurally stable. Also, we conducted the molecular dynamics simulations of the vaccine with TLR-3, because of the incorporation of the Human β-defensin 3 (hBD3) sequence at the N-terminus. This decision was informed by compelling research demonstrating the ability of hBD3 to augment the production of type I Interferon-β in response to the viral ligand mimic polyinosinic: polycytidylic acid (polyI: C) in both human and mouse primary cells [49]. Notably, this augmentation is intricately linked to TLR-3, a pivotal factor in antiviral innate immune responses. By targeting TLR-3 in our molecular docking, we evaluated our vaccine’s potential effectiveness, leveraging the known mechanism of action associated with hBD3 and its correlation to TLR-3-mediated immune responses. Last but not least, to determine the potency of the multi-epitope DNA vaccines as a final prevention option, more research utilizing *in silico* and in vivo patterns must be performed in future studies.

## Conclusions

In this study, a novel multi-epitope DNA vaccine against *Dabie bandavirus* was created using bioinformatics methods. More in vivo research is necessary, as well as testing on mouse models.

## Data Availability

No datasets were generated or analysed during the current study.
